# Nonnative Interactions in Coupled Folding and Binding Processes of Intrinsically Disordered Proteins

**DOI:** 10.1371/journal.pone.0015375

**Published:** 2010-11-04

**Authors:** Yongqi Huang, Zhirong Liu

**Affiliations:** 1 State Key Laboratory for Structural Chemistry of Unstable and Stable Species, College of Chemistry and Molecular Engineering, Peking University, Beijing, China; 2 Center for Theoretical Biology, Peking University, Beijing, China; 3 Beijing National Laboratory for Molecular Sciences, Peking University, Beijing, China; Indiana University School of Medicine, United States of America

## Abstract

Proteins function by interacting with other molecules, where both native and nonnative interactions play important roles. Native interactions contribute to the stability and specificity of a complex, whereas nonnative interactions mainly perturb the binding kinetics. For intrinsically disordered proteins (IDPs), which do not adopt rigid structures when being free in solution, the role of nonnative interactions may be more prominent in binding processes due to their high flexibilities. In this work, we investigated the effect of nonnative hydrophobic interactions on the coupled folding and binding processes of IDPs and its interplay with chain flexibility by conducting molecular dynamics simulations. Our results showed that the free-energy profiles became rugged, and intermediate states occurred when nonnative hydrophobic interactions were introduced. The binding rate was initially accelerated and subsequently dramatically decreased as the strength of the nonnative hydrophobic interactions increased. Both thermodynamic and kinetic analysis showed that disordered systems were more readily affected by nonnative interactions than ordered systems. Furthermore, it was demonstrated that the kinetic advantage of IDPs (“fly-casting” mechanism) was enhanced by nonnative hydrophobic interactions. The relationship between chain flexibility and protein aggregation is also discussed.

## Introduction

Deciphering how physical interactions affect protein behavior is fundamental to structural and functional biology. As a first approximation, interactions presented in the native state (native interactions) dominate in processes such as protein folding and binding, resulting in a funnel-like energy landscape with minimal frustration [1]–[3]. Under such conditions, the Gō-model [4], [5] has been widely adopted to generate valuable insights into protein folding and binding [6]–[10]. In realistic systems, however, the existence of nonnative interactions is inevitable. The effects of nonnative interactions on protein folding have been demonstrated in many experimental studies [11]–[23]. Nonnative interactions perturb the denatured state ensemble and thus affect the equilibrium stability [11], [12], lead to the accumulation of the on-pathway or off-pathway intermediate states [13]–[15], and most importantly, moderate the protein folding kinetics by perturbing the transition state [16], [17], [20], [21]. Nonnative interactions also act as a major driving force in the rapid collapse of an unfolded protein during the early stages of the folding process, which is important in preventing proteins from aggregating [22]. In the phosphorylation-activation process of a signal protein (nitrogen regulatory protein C), the disruption of some native contacts was compensated by the transient formation of nonnative interactions [23]. For protein binding processes, nonnative interactions have been recognized to be important in the initial formation of the non-specific encounter complexes, where long-range electrostatic interactions increase the diffusion process by the “steering effect”, and then short-range hydrophobic interactions facilitate the formation of the final specific complexes by a two-dimensional search on the surface [24]–[27].

The effects of nonnative interactions on protein folding have been extensively studied by simulations and analytical theories [28]–[45]. An all-atom simulation suggested that ∼20–25% of the energy in the transition state arose from nonnative contacts [28]. Nonnative hydrogen bonds in a simulation on the helix-coil transition were found to be most populated around the transition temperature [29]. Mutation can also change the population of nonnative contacts [30]. In general, the existence of nonnative interactions may influence protein stability and folding kinetics [31]. A lattice model simulation showed that nonnative interactions have little effect on protein stability, but would accelerate protein folding and thus give rise to the Φ-values that are negative or larger than unity [32]. Further detailed analytical and simulation studies found that the folding rate generally enhances initially as the nonnative interactions increase, but drops rapidly when the nonnative interactions are larger than a critical value [33]–[36]. The importance of nonnative interactions on folding kinetics was also validated by a direct comparison between simulation and experiment for the SH3 protein [37]. In the novel designed protein Top7 [46], it was revealed that the noncooperative folding kinetics is caused by both native topology and nonnative interactions [38], [39]. For proteins with more complicated topologies, e.g., knotted proteins, it was suggested that nonnative interactions play an essential role in the correct formation of the knots [40]. Despite the perturbation on folding kinetics, the protein folding mechanism is usually robust with respect to nonnative interactions [43], [44]. Compared with the extensive studies on protein folding, theoretical investigations on protein binding are relatively rare [47]–[51]. The mechanisms of the electrostatic rate enhancement via lowering the transition state energy and the dimensionality-reducing effect by non-specific binding to DNA and cell membranes are well understood; however, the effects of short-range hydrophobic interactions on general protein–protein binding processes remain unresolved.

The influences of nonnative interactions may be more prominent in intrinsically disordered proteins (IDPs). IDPs are a special family of proteins that lack unique tertiary structures under physiological conditions, either along the entire chain or in particular regions [52]–[55]. IDPs were predicted to be enriched in both prokaryotic and eukaryotic genomes [56], [57] and perform various functions, including transcription and translation regulation, cellular signal transduction, protein modifications, and molecular assemblies. In particular, IDPs have been shown to be associated with human diseases such as cancer, cardiovascular disease, amyloidosis and neurodegenerative diseases [58]. Although disordered when alone in solution, in many cases, IDPs undergo conformational transitions to folded states upon binding their biological targets to perform functions [59]. Gō-like models have been successfully applied to study the coupled folding and binding processes of IDPs [9], [49], [60]–[63]. This approach has contributed important insights into the characteristics of IDPs, e.g., the kinetic advantages in molecular recognition for IDPs through the “fly-casting” mechanism [64]. Considering the significant chain flexibility of IDPs, IDPs are expected to possess more nonnative interactions in the folding and binding processes than conventional ordered proteins. It would be important to investigate the different effects of nonnative interactions on IDPs and ordered proteins and whether taking into account the nonnative interactions would change the principles of IDPs elucidated by native-centric models.

In this paper, we conducted computer simulations to study how nonnative hydrophobic interactions affect the binding thermodynamics and kinetics of IDPs, and how these effects are related to the chain flexibility which distinguishes IDPs from the ordered proteins.

## Results

### IDPs are readily trapped into non-specific states

To investigate the effects of nonnative hydrophobic interactions on the coupled folding and binding of IDPs, we modified a coarse-grained Gō-like model of IDPs [62] to include a sequence-dependent hydrophobic-polar (HP) component which accounts for the nonnative hydrophobic interactions [37] (see *Materials and Methods*). We used our model to simulate the binding of the phosphorylated kinase-inducible domain (pKID) of the transcription factor cAMP response-element binding protein to the kinase inducible domain interacting domain (KIX) of the cAMP response-element binding protein. The pKID domain is a well characterized IDPs which folds upon binding to KIX [26], [65], [66]. In the model, a parameter *α* was introduced to scale the strength of the intra-molecular native interactions and thus tune the chain flexibility of pKID [62], while another parameter *K*_HP_ was used to describe the strength of the nonnative hydrophobic interactions.

**Figure 1:**
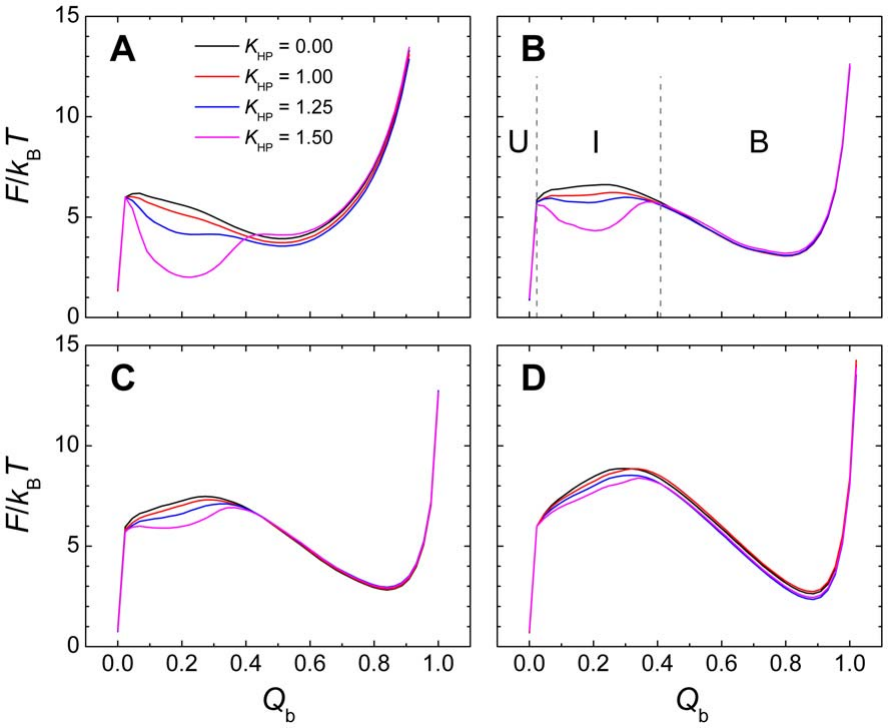
Free-energy profiles of the binding process. Free-energy profiles were calculated using the fraction of native intermolecular contacts (*Q*_b_) as a reaction coordinate for systems with different degrees of chain flexibility. (A–D) 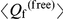 = 0.29, 0.46, 0.65, and 0.85 by tuning the intramolecular interaction parameter *α* from 0.1, 1.0, 1.5 to 3.0. The two vertical dashed lines in panel (B) indicate the definition of the unbound state (U), intermediate state (I), and bound state (B). The strength of the nonnative hydrophobic interactions (*K*_HP_) ranges from 0.00 to 1.50.

**Figure 2:**
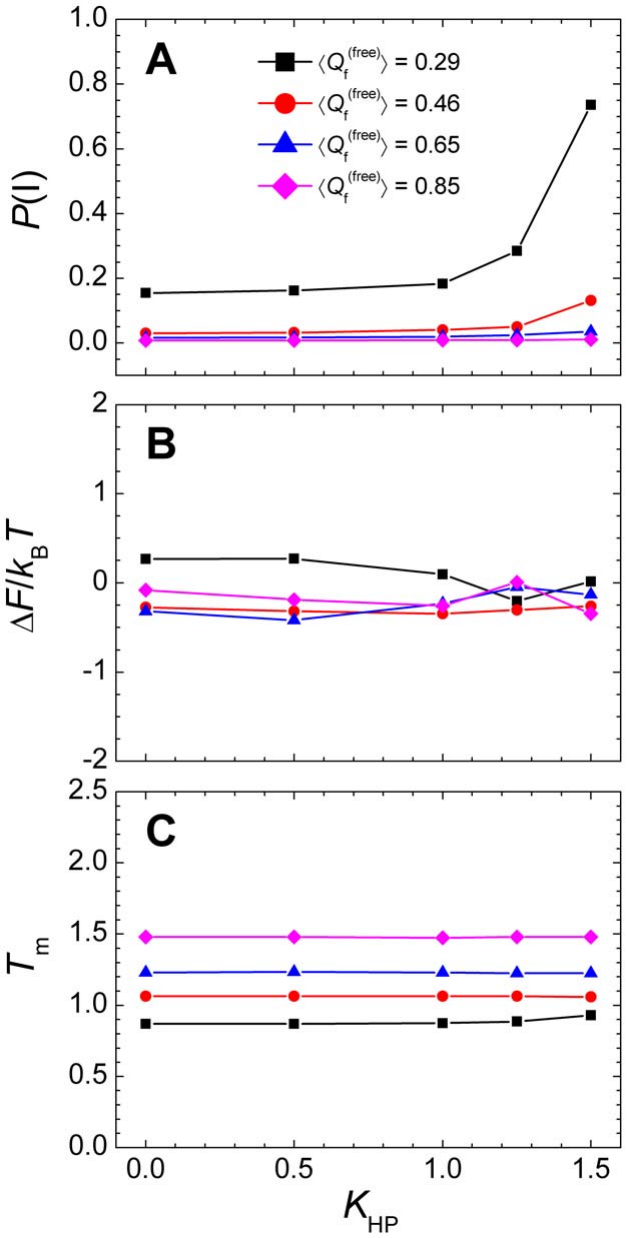
Thermodynamic properties of systems with different degrees of chain flexibility. (A) Correlation between the population of the intermediate state *P*(I) and the strength of the nonnative hydrophobic interactions, *K*_HP_. (B) Effect of the nonnative hydrophobic interactions on the stability of the complex which is measured by the free-energy difference between the bound state and the unbound state. (C) Effect of the nonnative hydrophobic interactions on the transition temperature *T*_m_.

The influences of nonnative hydrophobic interactions on the binding free energy of the pKID-KIX complex and their interplay with the chain flexibility are summarized in Figure 1. Here, we used the fraction of native contacts between the two proteins (*Q*_b_) as a reaction coordinate to depict the binding process. The nonnative hydrophobic interactions were found to mainly stabilize the partially bound states with moderate *Q*_b_ values, but had little influence on either the unbound or bound states. Over-strong nonnative hydrophobic interactions can even trap an intermediate state centered at *Q*_b_∼0.2 (Figures 1A,B). Interestingly, our results clearly showed that the effect of nonnative hydrophobic interactions on a disordered system (with a small 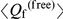) was more remarkable than that on an ordered system (with a large 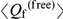). To quantitatively measure the influence on the equilibrium properties, we divided the conformation space into three states: the unbound state (U), intermediate state (I), and bound state (B) (Figure 1B). The population of the non-specific intermediate state increased as the strength of the nonnative hydrophobic interactions *K*_HP_ was increased, and the disordered system exhibited a more remarkable increase (Figure 2A). Although the nonnative hydrophobic interactions frustrated the binding free-energy landscapes, its influence on the stability of the complex was rather small because the free-energy difference between the bound and unbound states showed negligible change with *K*_HP_ (Figure 2B). When analyzing the transition temperature (*T*_m_, defined as the temperature corresponding to the peak in the heat capacity curve [62]), the same conclusion was reached: *T*_m_ remained constant with increasing *K*_HP_ (Figure 2C).

### Flexibility promotes dynamic and extensive non-specific interactions

**Figure 3:**
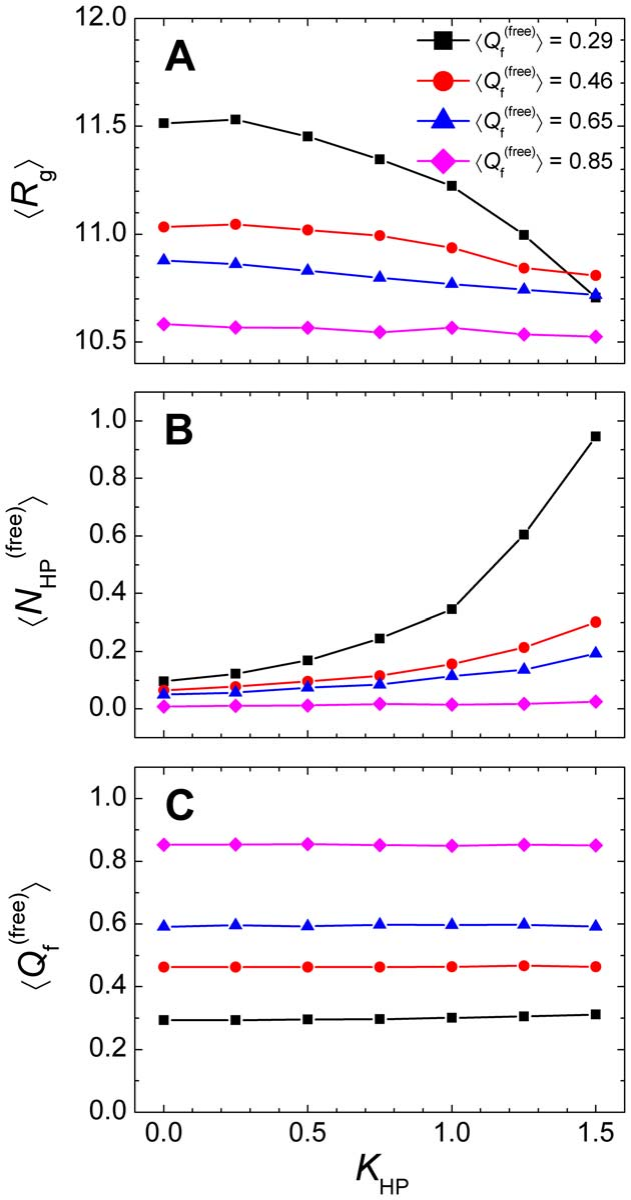
Properties of the free pKID domain in the presence of nonnative hydrophobic interactions. (A) radius of gyration (*R*_g_), (B) average number of nonnative contacts (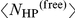), and (C) the average fraction of native contacts (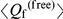).

**Figure 4:**
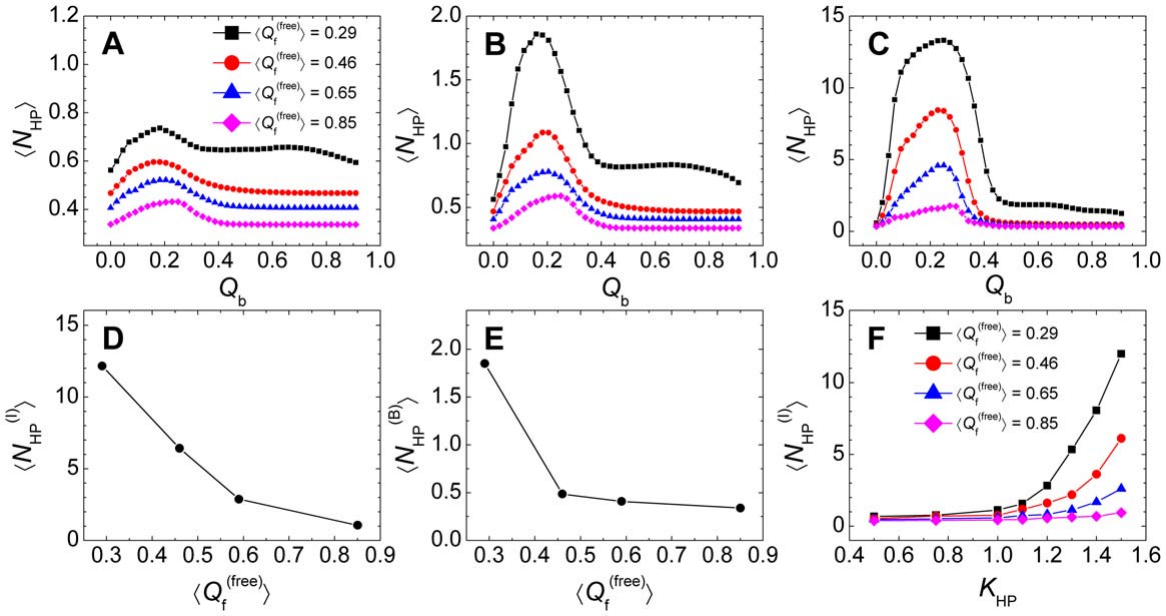
Characterization of the number of nonnative contacts. (A–C) The average number of nonnative contacts 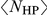 along the binding process when the strength of nonnative hydrophobic interactions was increased: (A–C) *K*_HP_ = 0.50, 1.00, and 1.50. (D) Correlation between the average number of nonnative contacts at the intermediate state 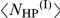 and 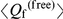. *K*_HP_ was set 1.50. (E) Correlation between the average number of nonnative contacts in the bound state 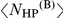 and 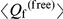. *K*_HP_ was set 1.50. (F) Correlation between 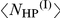 and *K*_HP_. The definitions of the intermediate state and bound state are presented in Figure 1.

To further characterize the nonnative hydrophobic interactions along the binding process, we examined the number of nonnative contacts (*N*_HP_) of pKID in the free state, binding intermediate state, and the bound state. In the free state, the radius of gyration (*R*_g_) showed that pKID underwent a minor compression in the presence of nonnative hydrophobic interactions (Figure 3A). Compared with the total number of intramolecular native contacts in the bound state (i.e., 25), the number of nonnative contacts was rather small (Figure 3B). For the most disordered system, 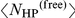 was only ∼1.0 even when *K*_HP_ = 1.5, and as expected, the ordered system showed no nonnative contacts. The small number of nonnative contacts observed for pKID in the free state explains the insensitivity of the equilibrium binding free-energy with respect to the strength of nonnative hydrophobic interactions (Figure 2B) because, by definition, the native bound state was affected only by native interactions. This observation was also consistent with results on protein folding which showed that the number of nonnative contacts of globular proteins is usually significantly smaller than the number of native contacts in the folded state [29], [39]. Therefore, intramolecular native contacts were not affected in the free state (Figure 3C). In contrast, in the binding process the intermediate state showed a considerable number of nonnative contacts, particularly for the disordered system (Figures 4A–C). Under the same strength of the nonnative hydrophobic interactions, the nonnative contact number of the intermediate state showed a remarkable decrease with decreasing chain flexibility (Figure 4D). Compared with the intermediate state, the bound state possessed fewer nonnative hydrophobic contacts (Figure 4E). This feature strongly indicates that chain flexibility promotes dynamic and extensive non-specific interactions during the binding process and will have a significant effect on the binding kinetics. When correlating the nonnative contact number of the intermediate state to the strength of the nonnative interactions, a sharp increase in the nonnative contact number appeared (Figure 4F), which leads to misbinding states (Figure 2A).

### Binding rate is accelerated by nonnative hydrophobic interactions

**Figure 5:**
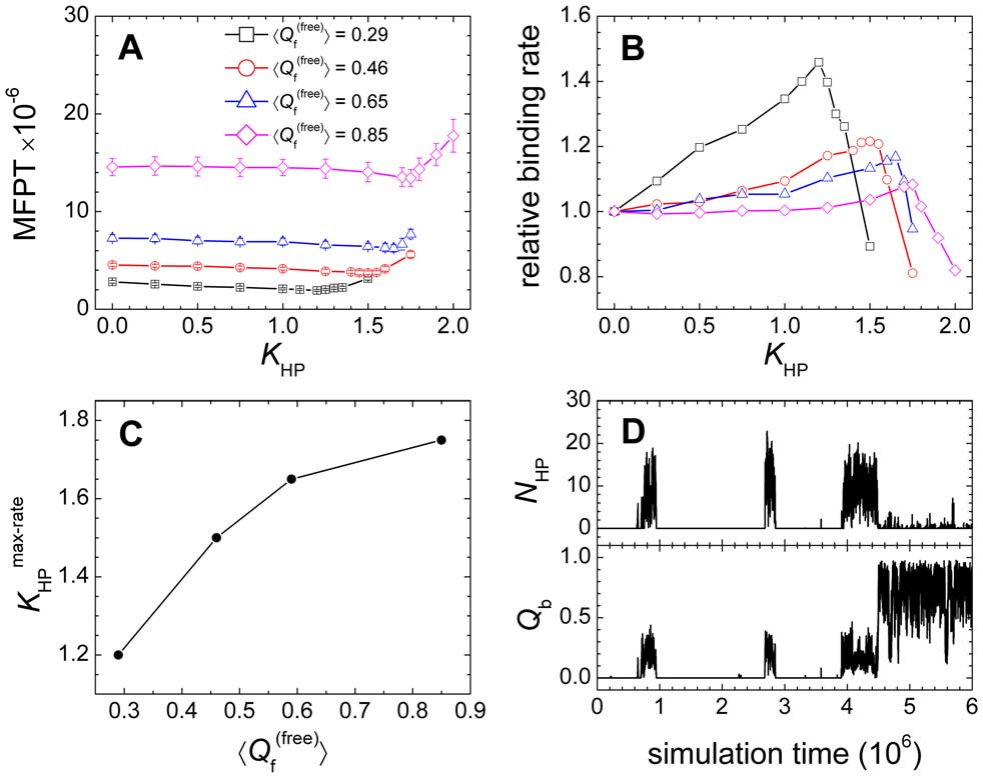
Effect of the nonnative hydrophobic interactions on the binding kinetics. (A) Mean first passage time (MFPT) of the binding process as a function of *K*_HP_. (B) Correlation between the relative binding rate and *K*_HP_. The relative binding rate was computed as 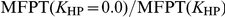. (C) Correlation between the *K*_HP_ corresponding to the maximum binding rate, *K*_HP_^max-rate^, and 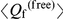. (D) A typical binding trajectory for the system with 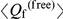 = 0.46 under *K*_HP_ = 1.50.

It was established from the Gō-models that IDPs possess faster binding rates than ordered proteins via the “fly-casting” mechanism which would facilitate molecular recognition [60], [62], [64]. Considering the findings in protein folding studies that nonnative interactions can accelerate or decelerate the folding rates [32]–[37], [39] and the above observation that nonnative contacts are more prevalent in IDPs than in ordered proteins, it would be intriguing to explore whether the kinetic advantage of IDPs will be smeared by the nonnative interactions. We simulated the binding kinetics of the pKID-KIX complex under various strengths of nonnative interactions, and the results are summarized in Figure 5. In a similar manner to protein folding, nonnative interactions have a nonlinear effect on the binding kinetics, i.e., the binding rate initially increased and then slowed sharply as the strength of the nonnative hydrophobic interactions increased, and this observation was independent of chain flexibility (Figures 5A,B). The existence of the critical strength of nonnative interactions was also observed in the work of Turjanski et al. [60]. In the binding-rate increase region, the disordered system possessed a greater binding rate than the ordered system, showing a kinetic advantage in the binding process even in the presence of nonnative hydrophobic interactions. As revealed from the free-energy analysis that the disordered system was more readily affected by nonnative hydrophobic interactions (Figure 1), the binding rate of the disordered system showed greater amplitude of change (Figure 5B). For the disordered system, the largest binding rate was 1.46 times as large as that when nonnative hydrophobic interactions were not included (*K*_HP_ = 0.0). However, for the ordered system, the largest binding rate was only 1.08 times as large as that at *K*_HP_ = 0.0. This indicates that the nonnative hydrophobic interactions further amplify the kinetic advantages of IDPs in the binding process. The most striking finding was that the strength of the nonnative hydrophobic interactions corresponding to the maximum binding rate (*K*_HP_^max-rate^) showed a strong dependence on the chain flexibility (Figures 5A, C). The disordered system exhibited a smaller *K*_HP_^max-rate^ than the ordered system. The reduction in the binding rate under strong nonnative hydrophobic interactions was caused by non-specific kinetic traps along the binding trajectory. Figure 5D exemplifies a binding trajectory of a system with 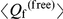 = 0.46 under *K*_HP_ = 1.50. Non-specific intermediate states are clearly shown. The chain flexibility dependence of *K*_HP_^max-rate^ indicates that, during the binding process, IDPs are more ready to form non-specific binding intermediates and even kinetically trapped misbinding states.

### The encounter complex is stabilized

**Figure 6:**
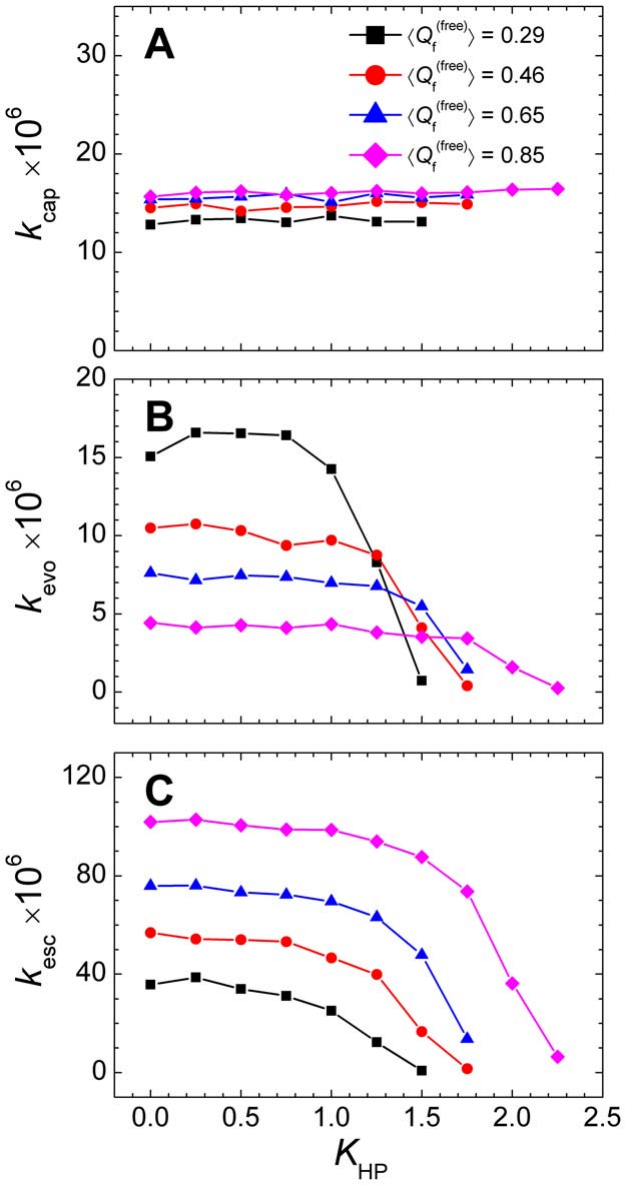
Kinetics analysis of the binding process. Effect of the nonnative hydrophobic interactions on (A) capture rate (*k*_cap_), (B) evolving rate (*k*_evo_), and (C) escape rate (*k*_esc_) for systems with various chain flexibilities.

The kinetic advantage of IDPs originates from the chain flexibility facilitating the encounter complex to evolve into the final binding complex rather than escape to the unbound state [62]. To investigate the influence of nonnative hydrophobic interactions on such a mechanism, we made an analysis by dissecting the binding process into a capture process and a further evolution process [62]:
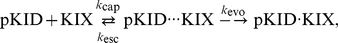
where pKID+KIX is the unbound state, pKID⋅KIX is the native bound state, while pKID⋅⋅⋅KIX is the loosely bound encounter complex state formed by the capture event. We considered an encounter complex occurred when the system evolved from an unbound state to a state with *Q*_b_>0 (usually had one intermolecular native contact). The effect of nonnative hydrophobic interactions on the capture rate *k*_cap_ was rather small for all systems and the disordered system possessed a slower capture rate (Figure 6A). Unlike the capture process, the evolving and the escape rates from the encounter state showed significant responses to the presence of the nonnative hydrophobic interactions (Figures 6B,C). Both Figures 6B and 6C show a two-stage profile, i.e., an initial plateau stage followed by a sharp decrease stage. The behaviors of the evolving and the escape rates as a function of the nonnative hydrophobic interaction strength were synchronic, but with opposite amplitude, namely, the disordered system (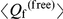 = 0.29) showed the greatest decrease of the evolving rate and the smallest decrease of the escape rate, whereas the ordered system (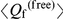 = 0.85) showed the smallest decrease of the evolving rate and the greatest decrease of the escape rate. Compared with Figure 5C, we also noticed that the points corresponding to the sharp decreases of the evolving and the escape rates were smaller than those corresponding to the sharp decreases of the overall binding rate.

The above results showed that the effect of the nonnative hydrophobic interactions on the binding process was primarily exerted on the evolution and escape stages. Unlike electrostatic interactions, which are long-range and accelerate the binding rate by the steering effect [24], nonnative hydrophobic interactions are short-range and their effects on the capture process is negligible (Figure 6A). However, in the encounter state, nonnative hydrophobic interactions contribute energetically to the stability of the encounter complex and so a reduction in the evolving and escape rates were observed (Figures 6B,C). Evolution and escape are two opposite processes in a binding event. Although increasing the nonnative hydrophobic interactions reduces the escape rate, it also reduces the evolving rate. Therefore, there is a balance between the escape and evolving rates to lead to the maximum binding rate.

### Influence of nonnative contact distance on nonnative hydrophobic interaction

**Figure 7:**
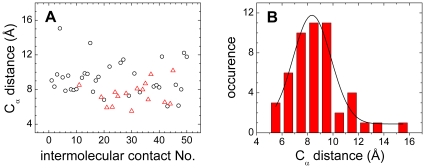
C_α_ distance distribution of native contacts in the pKID-KIX complex. (A) C_α_ distance distribution of native contacts formed by two hydrophobic residues (triangles) and others (circles). (B) C_α_ distance distribution of all native contacts.

**Figure 8:**
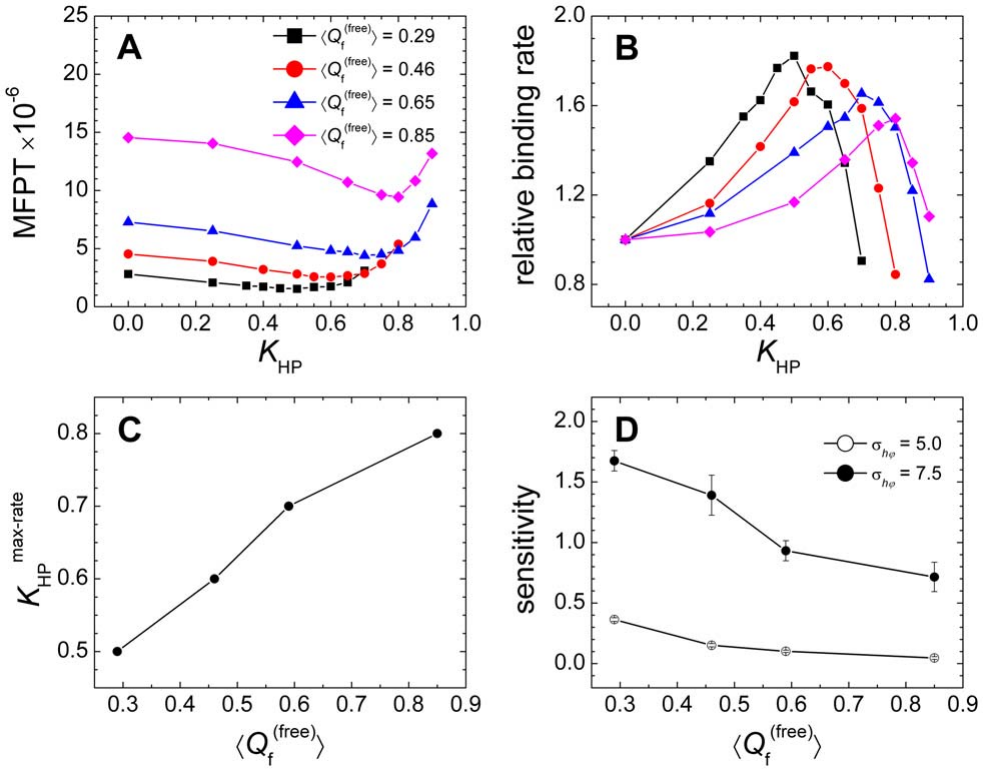
Effect of the nonnative contact distance on the binding kinetics. (A) Mean first passage time (MFPT) of the binding process as a function of *K*_HP_. (B) Correlation between the relative binding rate and *K*_HP_. (C) Correlation between the *K*_HP_ corresponding to the maximum binding rate *K*_HP_^max-rate^ and 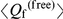. Contact distance σ*_hϕ_* = 7.5 Å was used in (A–C). (D) The sensitivity of the binding rate with respect to nonnative hydrophobic interactions for σ*_hϕ_* = 5.0 and 7.5 Å.

An adequate value for the nonnative hydrophobic contact distance (σ*_hϕ_*) is not well solved in the coarse-grained models. σ*_hϕ_* = 5.0 Å was adopted in Ref. [37], which is the same as the value used above. A slightly larger value, σ*_hϕ_* = 5.5 Å, was employed in Ref. [60]. Recently, a delicate scheme with adjustable σ*_hϕ_* values between 5.0 and 7.0 Å was applied to the designed protein Top7 [39]. By analyzing the C_α_ distance distribution of intermolecular native contact pairs (Figure 7), we found that a value of σ*_hϕ_* = 5.0 Å is positioned at the lower bound of the distribution. The average native contact distance between hydrophobic residues was 7.5 Å, which is about 1.8 Å smaller than that of other contacts. The effect of nonnative hydrophobic interactions on the binding process is dependent on the contact distance σ*_hϕ_* because lengthening σ*_hϕ_* will enhance nonnative hydrophobic interactions under the same interaction strength *K*_HP_. To confirm whether the σ*_hϕ_* value will alter the above findings that IDPs are likely to form nonnative contacts and their kinetic advantage is enhanced by nonnative hydrophobic interactions, we performed simulations with a larger σ*_hϕ_*, i.e., 7.5 Å. A similar nonlinear effect on the binding kinetics was observed (Figures 8A,B).

Increasing the contact distance σ*_hϕ_* to 7.5 Å enhanced the effect of nonnative hydrophobic interactions on the binding process (Figure 8B); meanwhile the *K*_HP_^max-rate^ value was reduced to a value smaller than or comparable to the strength of the native interactions (Figure 8C). To quantitatively describe the effect of σ*_hϕ_* on the binding rates, a linear fit in the regions where the binding rates were increasing in Figures 5B and 8B were performed, and the slope of the fit represented the sensitivity of the binding rates (Figure 8D). Under greater σ*_hϕ_* values, not only the binding rates of the disordered system is significantly affected, but also the binding rate of the ordered system is affected. However, Figure 8D shows that the binding rate of the disordered system was increased faster than that of the ordered system under different σ*_hϕ_* values.

## Discussion

Nonnative interactions are proved experimentally to play an important role in the encounter process and increase the binding rate by reducing the phase space by non-specific binding followed by a two-dimensional diffusion [24]–[26]. This mechanism is supported by our results that the nonnative hydrophobic interactions stabilize the encounter complexes (Figures 6B,C) and increase the binding rates (Figure 5A). More importantly, our results showed that the relative binding rate of the disordered system increased greater than that of the ordered system, thereby indicating that weak nonnative hydrophobic interactions further amplify the kinetic advantages of IDPs (Figure 5B) [62]. Electrostatic interactions have been shown to increase the “fly-casting” effect of IDPs [67]. From the viewpoint of interaction distance, electrostatic interactions are long-range and mainly affect the diffusion process, whereas nonnative hydrophobic interactions are short-range and mainly act on the two-dimensional searching process. For IDPs with short hydrophobic binding motifs flanked by charged regions, these two factors may combine to produce kinetic advantages over ordered proteins.

Our simulations provided important insights into the binding kinetics in the presence of nonnative hydrophobic interactions for systems with different chain flexibilities. Chain flexibility represents an important advantage to IDPs because such flexibility will facilitate the binding of IDPs to several targets [68], [69] with greater binding rates [62], [64]. However, nonnative hydrophobic interactions can also hamper specific binding of IDPs to target proteins because of kinetic traps. In our simulations, a disordered chain was found to be more readily trapped into misbinding states (Figures 1,5,8), possibly because the disordered chain lacks structural constraints to prevent misbinding [70]. Structural inspection of snapshots from simulations gave non-specific binding states consistent with those in Ref. [60]. Although hydrophobic residues are sparsely found in IDPs, the interface of complexes formed by IDPs often have more hydrophobic–hydrophobic contacts than protein complexes formed by ordered proteins [71]. Consequently, IDPs possess a misbinding potential. Compared with the specific binding state, misbinding states may be less stable and as shown by Vavouri et al., the off-target binding is tightly regulated through the control of the concentration of IDPs [72].

Although simple, the model adopted in this work allowed us to isolate the effect of chain flexibility and provided insights into the relationship between chain flexibility and protein misbinding/aggregation. These findings support the importance of structural constraints in preventing aggregation [70], [73] and structural flexibility in binding promiscuity [72], and also provide new clues to regulate protein-protein interactions through controlling the flexibility of proteins or binding motifs.

In conclusion, in this work, we introduced nonnative hydrophobic interactions into the Gō-like model to investigate how they affect the binding process. Using the KIX-pKID complex as a model system, we continuously tuned the chain from a disordered to an ordered form to characterize the interplay between chain flexibility and nonnative interaction effects. The results showed that the free-energy profiles became rugged and the intermediate states occurred when nonnative hydrophobic interactions were introduced. The binding rate initially accelerated and then dramatically decreased as the nonnative hydrophobic interaction strength was increased. Both thermodynamic and kinetic analysis showed that the disordered system was more readily trapped into non-specific misbinding states than an ordered system. This supports the idea that IDPs are prone to form promiscuous interactions and aggregate. Furthermore, our results showed that weak nonnative hydrophobic interactions amplify the kinetic advantages of IDPs in specific binding processes.

## Materials and Methods

### Protein structure and quantities describing the coupled folding-binding process

The protein complex used in this study is formed by the phosphorylated kinase-inducible domain (pKID) of the transcription factor cAMP response-element binding protein (CREB) and the kinase-inducible domain interacting domain (KIX) of the CREB binding protein [26], [65], [66]. The pKID domain (Asp119–Pro146) is disordered in the free form and folds into two α-helices upon binding to the structured KIX domain [26]. The native contact set was built based on the CSU software [74]. The fraction of native intramolecular (folding) contacts, *Q*_f_, was used to monitor the folding process, and the fraction of native intermolecular (binding) contacts, *Q*_b_, was used to monitor the binding process. The average fraction of intramolecular native contacts of pKID in its free form, 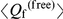, was used to quantify the degree of disorder (chain flexibility) of the model.

### Modified native-centric Gō-like model with nonnative hydrophobic interactions

In this work, we modified a native-centric continuum Gō-model with coarse-grained C_α_ chain representation [7], [62], [75] to include nonnative hydrophobic interactions [37]. In the model system (pKID-KIX complex), the KIX domain was the ordered target and kept frozen during the simulations, whereas the pKID domain was free and tuned from a disordered to an ordered form by increasing the intramolecular interaction strength [62]. Thus, the total potential energy including nonnative hydrophobic interactions is proposed as:
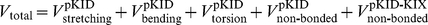
where
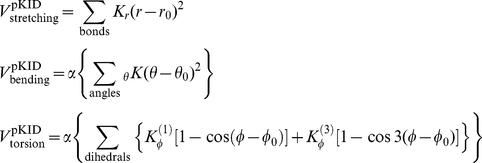
Compared with Ref. [62], *α* is also introduced into the bending and torsion terms to better control the chain flexibility. Non-bonded interactions were divided into native interactions, excluded volume repulsions, and nonnative hydrophobic interactions:
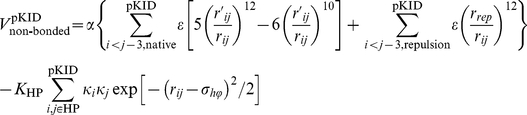


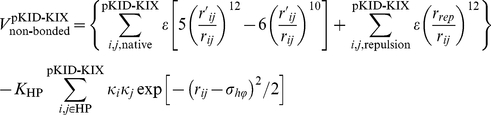

*r*, *θ*, 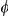, and *r_ij_* were the virtual bond length, bond angle, torsion angle, and non-bonded spatial distance defined by the C_α_ atoms, respectively; *r*_0_, *θ*_0_, 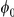, and 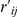 were the corresponding native values available from the PDB structure (PDB code for the pKID-KIX complex is 1KDX [66]). Non-bonded interactions were only considered when two C_α_ atoms *i* and *j* were separated sequentially by at least three residues within one chain (the pKID domain) or when they came from different chains. For native interactions, a 12-10 Lennard-Jones (LJ) form potential was used; whereas for nonnative interactions, *r_rep_* parameterized the excluded volume repulsion between residue pairs that do not belong to the given native contact set. The nonnative hydrophobic interaction term adopted here has been used to account for the nonnative hydrophobic interactions present in protein folding processes [37]. Alanine, valine, leucine, isoleucine, phenylalanine, methionine, tryptophan, and tyrosine were considered as hydrophobic amino acids. We did not distinguish between hydrophobic residue types, so *κ_i_* was the same for all hydrophobic residues and set at 1.0. The overall strength of the hydrophobic interactions was controlled by *K*_HP_. We adopted σ*_hϕ_* = 5.0 Å as in Ref. [37] to control the hydrophobic interactions, and also tested the influence of the σ*_hϕ_* value (see *Influence of nonnative contact distance on nonnative hydrophobic interaction*). The summation in the nonnative hydrophobic interaction term excludes hydrophobic pairs in the native contact because they have already been included in the native interactions. Other parameters were set *r_rep_* = 4.0 Å, *K_r_* = 100 *ε*, *K_θ_* = 20 *ε*, 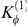 = *ε*, 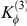 = 0.5 *ε*. The interaction strength was controlled by the parameter *ε*, which was fixed at 1.0 in this study. The parameter *α* scaled the intramolecular interactions within the pKID domain and tuned the degree of chain disorder (flexibility): 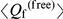 was calculated to be 0.29, 0.46, 0.59 and 0.85 for *α* = 0.1, 1.0, 1.5, and 3.0, respectively.

### Thermodynamics and kinetics simulations

Simulations were performed by Langevin dynamics in an over-damped region, with a friction constant of 0.1 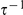 (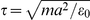), where the length scale *a* was set to 4 Å, the mass *m* was set to 1.0, and the reference energy scale 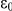 was 1.0 as in Ref. [76], [77]. The molecular dynamics time step was set to 0.005 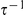. Simulation temperatures were chosen to be the transition temperature of the binding process, i.e., the temperature where the system has equal probability in the bound state and the unbound state, when the nonnative hydrophobic interaction strength *K*_HP_ was set to zero. Other parameters were set as in Ref. [62].

A pKID chain and a KIX chain were put in a 200 Å cubic box with periodic boundary conditions. The KIX domain was kept frozen at the box center while the pKID was free to move. A high temperature unbinding simulation was performed to provide 400 randomly chosen unbound conformations. An unbound conformation was defined by the fraction of native contact *Q*_b_ = 0 and a mass-center distance between the two proteins *ΔR*>45 Å. Subsequently, 400 binding simulations were performed starting from 400 unbound structures. A bound state was considered to occur when the system reached the minimum of the free energy as in Ref. [62]. The encounter state was reached when the system evolved from an unbound state to a state with *Q*_b_>0 (usually have one native contact). Kinetic data were averaged from the resulting trajectories.

By dissecting a binding trajectory into an encounter step, an escape step, and an evolution step, we accumulated the transition number (*N*) and the averaged transition time (measured by the mean passage time, MPT) between any two states. The escape rate *k*_esc_ and the evolving rate *k*_evo_ were calculated as:
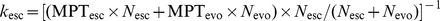


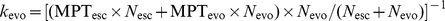
where MPT_esc_ and MPT_evo_ are the mean passage time from the encounter state to the unbound state and from the encounter state to the bound state, respectively; *N*_esc_ and *N*_evo_ are the corresponding numbers of transitions. The capture rate was calculated as 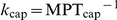; MPT_cap_ is the mean passage time from the unbound state to the encounter state.

The bias potential and the histogram technique were used for conformational sampling [78], [79]. The free energy was calculated as 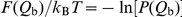, where *P*(*Q*_b_) is the normalized population distribution as a function of *Q*_b_.
